# When the Tree Branch Affects the Fruits: A Case of Celiac Artery Dissection

**DOI:** 10.7759/cureus.56695

**Published:** 2024-03-22

**Authors:** Edwin R Mosquea Gomez, Bijal Mehta

**Affiliations:** 1 Internal Medicine, Hackensack Meridian Mountainside Medical Center, Montclair, USA

**Keywords:** left abdominal pain, ischemic infarcts, arterial dissection, splenic infarcts, celiac dissection

## Abstract

Arterial dissection is a laceration of an artery or arterial bed, that can extend to contiguous arteries and lead to accumulation of blood providing a great risk for thrombi formation, and possible ischemic events. Celiac artery dissection is a very rare pathology, with an unknown prevalence and a pathophysiology that still needs to be elucidated. Diagnosis has increased in the last decade due to higher imaging modalities and accessibility of such that provide simpler identification, as well as which treatment should be applied to a particular patient.

In this case report, we present a 44-year-old male with abdominal pain, found to have on computed tomography angiography (CTA) a dissection of the celiac artery with extension to the splenic artery, causing multiple splenic infarcts, demonstrating that such lesions can be the cause of unexplained thrombosis in a certain patient population. Due to its complex presentation, management can differ based on the characteristics of the dissection as well as organ involvement, these modalities range from anticoagulation to surgical or endoscopic intervention. This case highlights the rare occurrence of an isolated dissection at a visceral artery causing thrombosis in a relatively healthy patient.

## Introduction

The celiac trunk is a branch of the descending aorta that supplies the abdominal esophagus, stomach, duodenum, liver, gallbladder, spleen, and pancreas. A dissection is a tear of the internal lining, regarded as a gradual process [1,2]. Celiac artery dissection is a very rare pathology with pathophysiology still to be elucidated [2,3]. Diagnosis can be an incidental finding on imaging and is sometimes associated with a clinical syndrome consisting mostly of abdominal pain depending on the organs affected [4,5]. Treatment consists of anticoagulation and even surgical intervention [6-8]. We present a case of a young patient with no significant medical history who experienced abdominal pain, was diagnosed with splenic infarcts and later discovered to have celiac-splenic artery dissection as the underlying cause.

## Case presentation

A 44-year-old male with no significant past medical history presented with a three-month history of chronic left upper quadrant abdominal pain. Family history was significant for hypertension in both parents, but not the patient nor any other risk factor for arterial weakening. Vital signs were normal on admission. Physical examination was remarkable for left upper quadrant abdominal tenderness. Blood work resulted in a normal comprehensive metabolic panel, cell blood count, coagulation parameter, and lipid panel, but positive dilute Russell's viper venom time (DRVVT) as well as factor 8 activity, aiding in thrombi formation. Abdominal computer tomography (CT) showed multiple splenic infarcts without evidence of stenosis or aneurysm to account for such distal embolization. Transthoracic echocardiography revealed a normal left ventricular size, wall thickness, and systolic function, with an ejection fraction of 60-65%. The right ventricle was of normal size; normal valve without vegetations and trace mitral regurgitation. A portal vein venous doppler showed a patent hepatic vasculature with normal flow in the portal vein and branches, but hypoechoic heterogeneous areas in the mid to inferior aspect of the spleen were noticed. The patient was started on anticoagulation with a full dose of enoxaparin and transitioned to oral anticoagulant at a later time before discharge. 

**Figure 1:**
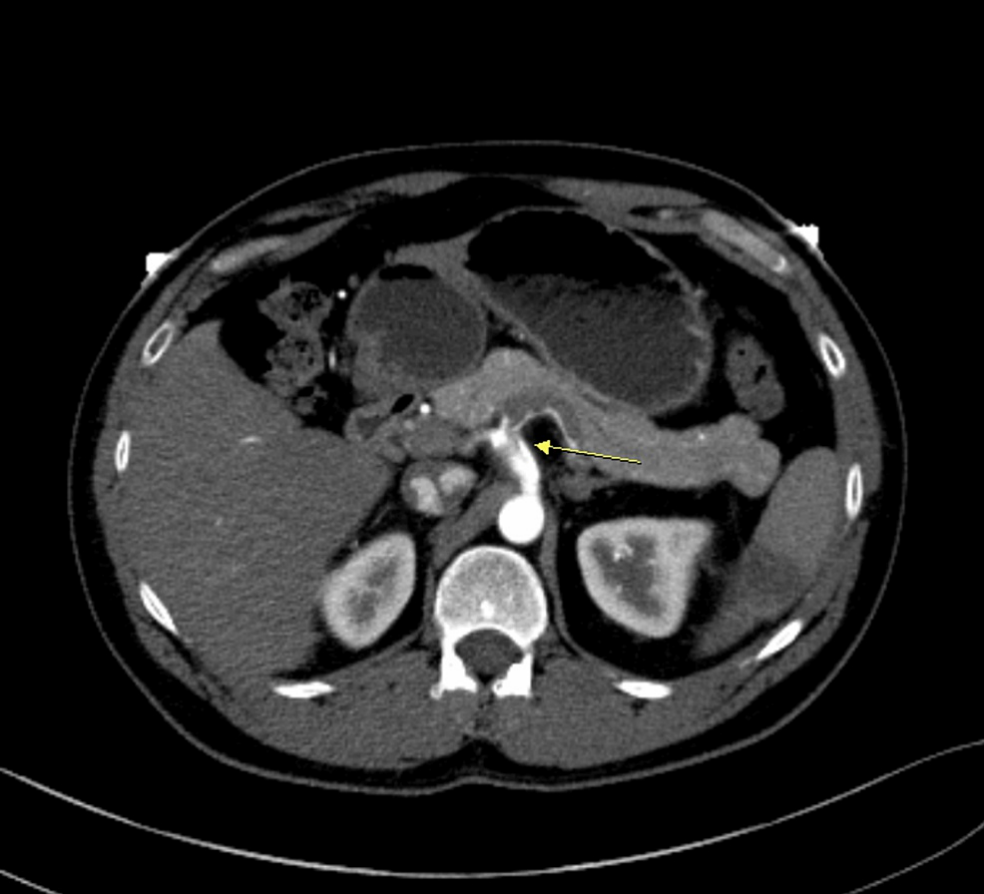
Celiac artery dissection (arrow), splenic infarct (coronal plane). Celiac artery dissection extending to irregular and stenotic splenic artery, along with hypodensity of spleen suggestive of ischemia/infarcts.

**Figure 2:**
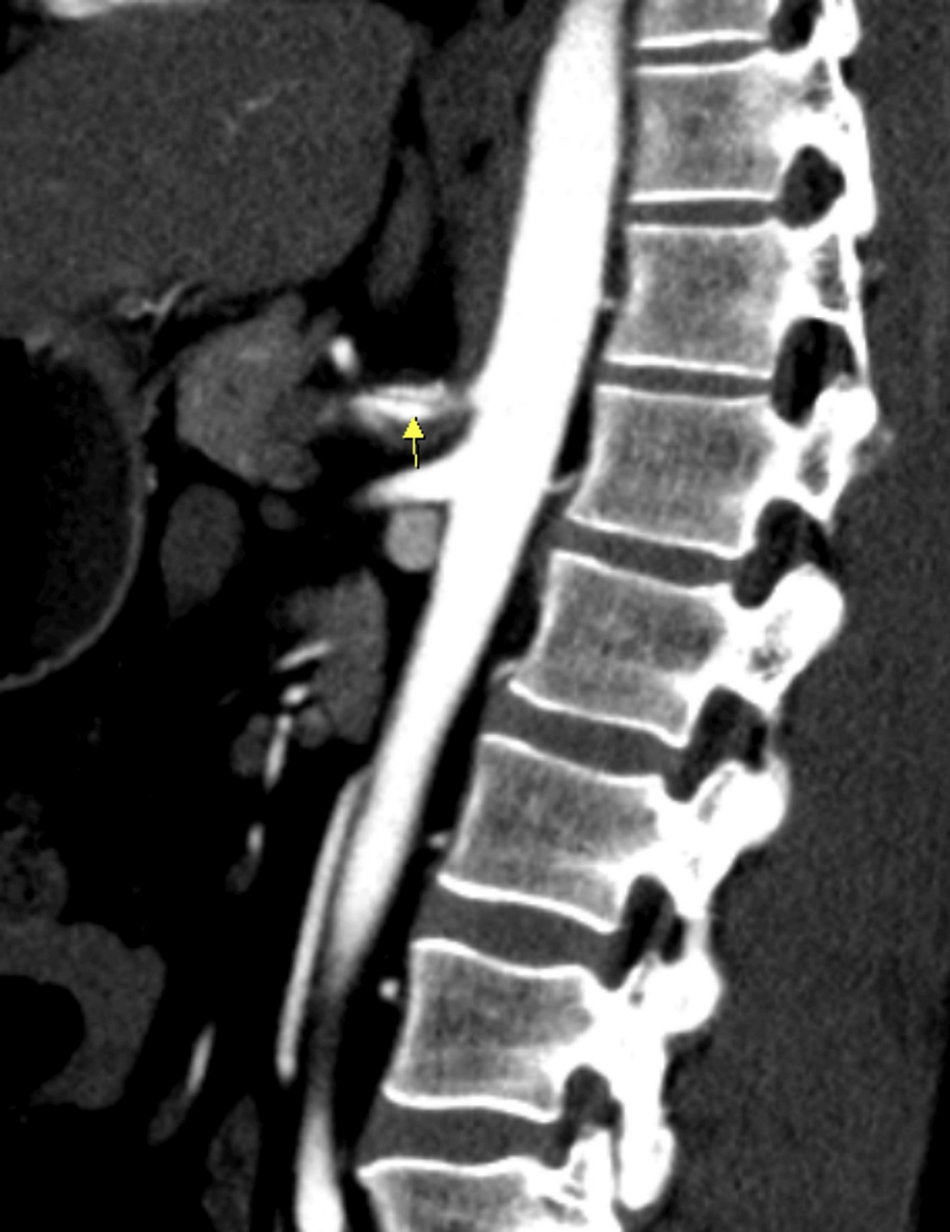
Celiac artery dissection (arrowhead) (sagittal plane).

**Figure 3:**
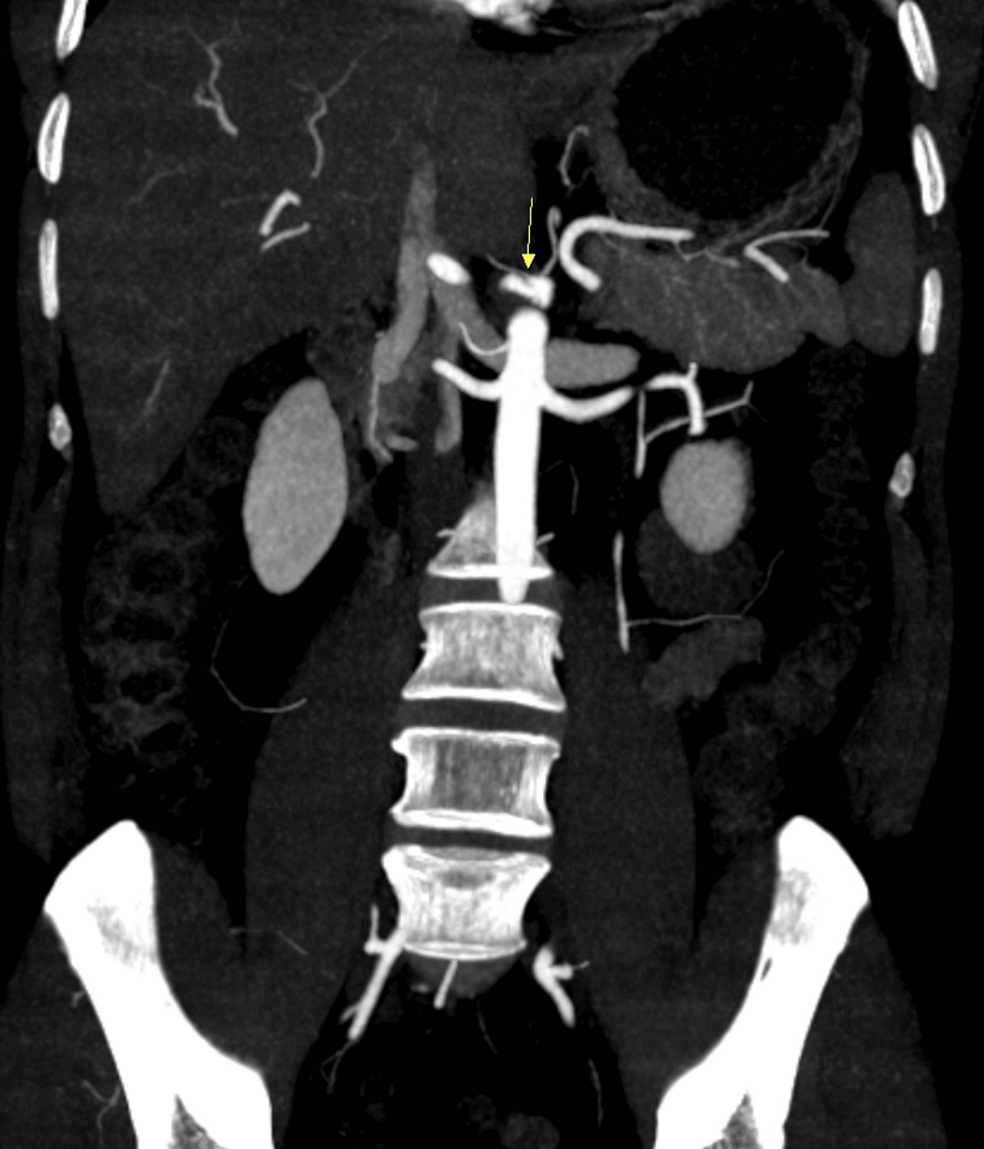
Celiac artery dissection (arrow) (frontal plane).

CT angiography was done to rule out secondary causes that could have led to infarction of the spleen, noticing celiac artery dissection that extended into the splenic artery (Figures 1, 2, 3).

Vascular surgery determined no acute need for surgical intervention and recommended continuing anticoagulation for three to six months given the stability of the splenic lesion. 

## Discussion

The celiac trunk is the second aortic branch that supplies many organs in the abdominal cavity, with its three main branches: left gastric, splenic, and common hepatic artery.

An arterial dissection is a tear of the internal lining of an artery that creates a false lumen, causing narrowing or even closing the artery completely and leading to an accumulation of pooled blood [1-10]. This may occur spontaneously or associated with trauma, with an incidence of five to 30 cases per million population [2]. Spontaneous dissection of visceral arteries was first described by Bauersfeld in 1947, followed by the first description of spontaneous celiac artery dissection in 1959, with only 13 cases described by 2001 [5,11].

Celiac artery dissection is more common in males (5:1 ratio), with average age of diagnosis at 55 years [3,4,8]. No clear etiology has been found; however predisposing risk factors include hypertension, pre-existing vascular disease, pregnancy, cystic medial necrosis, abdominal aortic aneurysm, fibromuscular dysplasia, smoking, trauma and connective tissue disorder, leading to arterial wall weakening, which our patient did not have [3,4,9]. Most patients are asymptomatic, however when patients present with symptoms the most common is abdominal pain (71%), followed by back pain (8%) and nausea (4%) [4,10], likely due to involvement of splenic, renal, or superior mesenteric arteries causing infarction or ischemia [5].

Diagnosis is made via imaging, with computed tomography angiography (CTA) considered the primary technique for diagnosing celiac artery dissection, due to being quick and easily accessible [4,7,9], magnetic resonance angiography, conventional angiography can be used as an alternative and color duplex sonography being an excellent choice for patients in whom kidney function might be compromised; however, it has its own limitation since it is not as detailed as CTA [4,6,9].

Even though there is no consensus on management, it consists of anticoagulation therapy with heparin, warfarin, or direct oral anticoagulant, with a duration of up to six months. Some patients require surgical intervention due to the presence of fusiform or sacculated aneurysms, occlusive lesions jeopardizing the lower digestive tract, or arterial complications such as liver ischemia [7,8].

## Conclusions

Arterial dissection is one of the diagnoses that can be missed if it is not present in the mind of the clinician, especially when the arteries involved do not necessarily cause obvious symptomatology or hemodynamic compromise. Celiac artery dissection is a very rare pathology, with increasing diagnosis due to the advances in imaging. There are many predisposing factors to develop a dissection that can ultimately lead to thrombi formation placing the patient at risk for intra-abdominal ischemic events; however, in many cases, its cause still cannot be elicited. This case highlights the importance of diagnosis of visceral dissection, management, and determination of which candidates require surgical intervention. Recognition of this pathology is crucial for appropriate risk stratification and conservative versus surgical management.
